# Computer Generated Holography with Intensity-Graded Patterns

**DOI:** 10.3389/fncel.2016.00236

**Published:** 2016-10-17

**Authors:** Rossella Conti, Osnath Assayag, Vincent de Sars, Marc Guillon, Valentina Emiliani

**Affiliations:** Wave Front Engineering Microscopy Group, Neurophotonics Laboratory, Centre National de la Recherche Scientifique, UMR 8250, University Paris DescartesParis, France

**Keywords:** microscopy, computer generated holography, optogenetics, graded intensity holograms, ChR2 photostimulation

## Abstract

Computer Generated Holography achieves patterned illumination at the sample plane through phase modulation of the laser beam at the objective back aperture. This is obtained by using liquid crystal-based spatial light modulators (LC-SLMs), which modulate the spatial phase of the incident laser beam. A variety of algorithms is employed to calculate the phase modulation masks addressed to the LC-SLM. These algorithms range from simple gratings-and-lenses to generate multiple diffraction-limited spots, to iterative Fourier-transform algorithms capable of generating arbitrary illumination shapes perfectly tailored on the base of the target contour. Applications for holographic light patterning include multi-trap optical tweezers, patterned voltage imaging and optical control of neuronal excitation using uncaging or optogenetics. These past implementations of computer generated holography used binary input profile to generate binary light distribution at the sample plane. Here we demonstrate that using graded input sources, enables generating intensity graded light patterns and extend the range of application of holographic light illumination. At first, we use intensity-graded holograms to compensate for LC-SLM position dependent diffraction efficiency or sample fluorescence inhomogeneity. Finally we show that intensity-graded holography can be used to equalize photo evoked currents from cells expressing different levels of chanelrhodopsin2 (ChR2), one of the most commonly used optogenetics light gated channels, taking into account the non-linear dependence of channel opening on incident light.

## Introduction

Originally demonstrated for multi-trap optical tweezers (Curtis et al., 2002), computer generated holography (CGH) has been recently proven to be an efficient approach for patterned voltage imaging (Foust et al., 2015) and optical control of neuronal activity using caged compounds (Lutz et al., 2008; Nikolenko et al., 2008; Daria et al., 2009; Dal Maschio et al., 2010; Anselmi et al., 2011; Yang et al., 2011, 2014) or optogenetics molecules (Packer et al., 2012, 2014; Bègue et al., 2013; Papagiakoumou, 2013; Reutsky-Gefen et al., 2013). Briefly the principle of CGH consists in computing, with an iterative algorithm, the phase pattern at the rear aperture of the microscope objective that reproduces the desired target intensity in the objective focal plane. The calculated phase-hologram is addressed to a liquid crystal matrix spatial light modulator (LC-SLM) that is designed to impose the phase modulation onto the input laser beam's wavefront. After propagation through the objective, the beam is focused onto an intensity pattern, reproducing straight away the desired template. In previous demonstrations, computer generated holograms have been calculated based on binary input images representing the target spatial distribution. By doing so, the calculated phase hologram generates a binary light distribution. Here we show that using graded input images enables generating graded intensity holograms. We demonstrate this approach in three different applications. In a first one graded-intensity holograms are used to compensate for the position dependent diffraction efficiency of the Liquid Crystal matrix thus achieving uniform light distribution across the entire field of view. Secondly we demonstrate that intensity-graded holograms can be used to rescale the illumination light distribution on the base of the fluorescence emission of the sample. Finally we demonstrate the use of intensity-graded holograms to equalize optogenetics evoked photocurrents from cells with different expression levels.

## Materials and methods

### Cell culture

Hamster Chinese Ovary's cells were cultured in an incubator at 32°C and 5% CO2 in a D-MEM/F12 GlutaMAX medium (Life Technologies) with the addition of 1 mM glutamine, 1% streptomycin and 10% fetal bovine serum. Cells were plated on Thermanox plastic coverslips (Thermo Scientific) 24 h prior to transfection. The DNA was transfected using the EX-Gen 500 transfection reagent and cells were recorded 24–48 h after transfection. The plasmid used was a cell filling type, which contains a ribosomal skip site (p2A) that mediates a co-translational cleavage event resulting in the release of the photochannel and the YFP marker individually. (Osborn et al., 2005)(AAV-hSyn-hChr2 (H134R)-p2A-eYFP; kindly provided by Karl Deisseroth).

We have chosen to use passive cells to test our technique to avoid the contribution of other endogenous channels to the signal. In particular we used CHO cells because of the absence of gap junctions (see Supplementary Figure 1) which can generate spurious contributions to the electrical response from neighboring cells.

### Imaging

Cultured cells were transferred for recording in a chamber mounted on the head stage of an upright microscope (Axio Examiner, Zeiss) and monitored through a 63x water immersion objective under transmitted IR light by means of a Cool Snap ES2 cooled CCD camera (Photometrics). Transfected cells were identified under epi-fluorescence illumination. A Mercury Arc Lamp (Lumen Dynamics) with a FITC filter set (Semrock filter set: 482/35 nm excitation, 536/40 emission filter) was used to excite the YFP present in the cells and thus detect the levels of expression. All fluorescence images were taken with the same exposure time (200 ms) and lamp settings.

### Photostimulation

**Figure 1:**
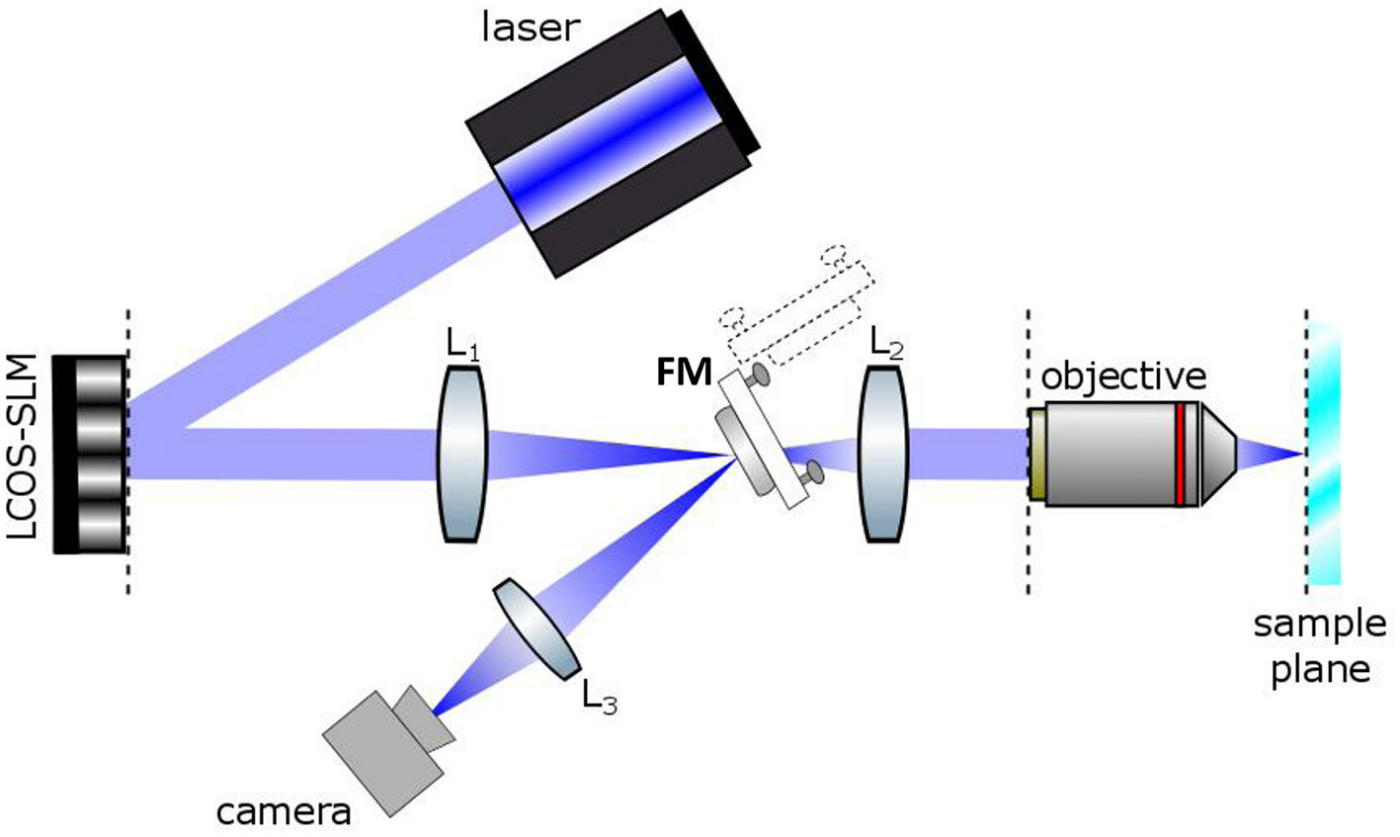
**Basic scheme of the Computer Generated Holographic system**. A 405 nm laser beam illuminates a spatial light modulator (LCOS-SLM). The SLM is imaged onto the back focal plane of the objective lens by a Galileo telescope made of lenses L_1_ and L_2_. A camera is located in an intermediate image plane where the holographic images can be alternatively directed by a flip mirror (FM).

The optical set up for holographic light stimulation is schematized in Figure 1. Briefly, the beam of a commercial laser (λ = 405 nm; CNI-Laser MLL-III-405) is expanded (3x) and conveyed to a liquid crystal Spatial Light Modulator (LC-SLM) (LCOS Hamamatsu model X10468-05; refreshing rate 60 Hz) which is addressed with holographic phase profiles calculated by a custom made interface (Wavefront Designer IV) based on the iterative Gerchberg and Saxton (GS) algorithm (Wyrowski and Bryngdahl, 1988). The modulated light at the exit of the SLM is then refocused on the focal plane of the objective through a telescope (L_1_, f_1_ = 300 mm; L_2_, f_2_ = 165 mm). The magnification of the telescope is chosen in order to match the SLM short axis with the diameter of the objective back aperture (Zeiss, 63X W 1 NA). The holographic images can be displayed on a camera located at an intermediate image plane, using a flip mirror. Photostimulation trigger and duration were controlled by the pClamp software (Molecular Devices). The intensity of the photostimulation was controlled manually with a half wave plate.

### Electrophysiology

Recordings were performed at room temperature 24–48 h after transfection. The extracellular medium contained: NaCl 140 mM, KCl 5 mM, CaCl2 2 mM, MgCl2 1 mM, Hepes 20 mM, Glucose 25 mM. PH was adjusted to 7.5. Electrophysiological recordings were performed through a Multiclamp 700B Amplifier (Molecular Devices) in the whole-cell voltage clamp recording configuration. Patch pipettes, pulled from borosilicate glass capillaries, had a resistance in the bath that ranged from 4 to 6 MΩs. The intracellular solution contained: KCl 140 mM, MgCl2 2 mM, Mg ATP 2 mM, Na GTP 0.4 mM, Hepes 10 mM, BAPTA 20 mM. PH was adjusted to 7.3; osmolarity 330. Cells were maintained at -40 mV throughout recordings. The holding current ranged from 0.8 to 80 pA.

### Data acquisition and analysis

Electrical data were filtered by the Multiclamp 700B amplifier and digitized through a Digidata 1440A DAC converter controlled by pClamp 10.3 software, which was also used for triggering the mechanical shutter (Uniblitz) of the laser. Short (100 ms) hyperpolarizing steps (5 mV) were used to measure access resistance which ranged from 8 to 20 MΩ and membrane resistances varying from 200 MΩ to 12 GΩ. Evoked photocurrents were triggered by short opening (from 15 to 160 ms) of a mechanical shutter in front of the laser controlled by pClamp after a short baseline period (100 ms). The filter frequency was 10 KHz for resistance steps recordings and 6 KHz for ChR2 current recordings. Sampling frequencies were of 20 KHz and 10 KHz respectively.

For analysis, the data were imported into the Igor programming environment (Wavemetrics) and analyzed using home-made analysis routines.

Epi-fluorescence illumination and image acquisitions were controlled by the image acquisition software SlideBook 6 (Intelligent Imaging Innovations). Images were then saved in TIFF format and uploaded into the home made Wavefront Designer IV software for analysis of the level of expression and generation of the correct pattern to be sent to the SLM. The average fluorescence was calculated as the integral over the whole cell divided by its surface.

All other analysis and statistics were performed into the Igor programming environment. Results are given as mean ± one standard deviation (SD) or standard deviation of the mean (SEM) as noted in the text.

## Results

### Holograms with graded intensity

As a first test of our capability to control the distribution of light intensity, we used graded intensity holograms GI-Holograms to compensate for the position–dependent-diffraction efficiency of LC-SLM.

The LC-SLM used to modulate the phase of the incident laser wavefront is a pixelated structure of finite size: each liquid crystal on the matrix of the SLM is a pixel and the total number of pixels is finite. Thus, the LC-SLM acts as a blazed phase grating, the steps of the blaze being the different phase values used for modulation of the incident wavefront. The efficiency of an SLM is defined by its capacity to diffract the incident light in the 1st order of diffraction and is given by the ratio of the light diffracted in the 1^st^ order over the incident light on the SLM: $\delta_{1st}=\frac{I_{1storder}}{I_{\text{i}ncident}}$.

**Figure 2:**
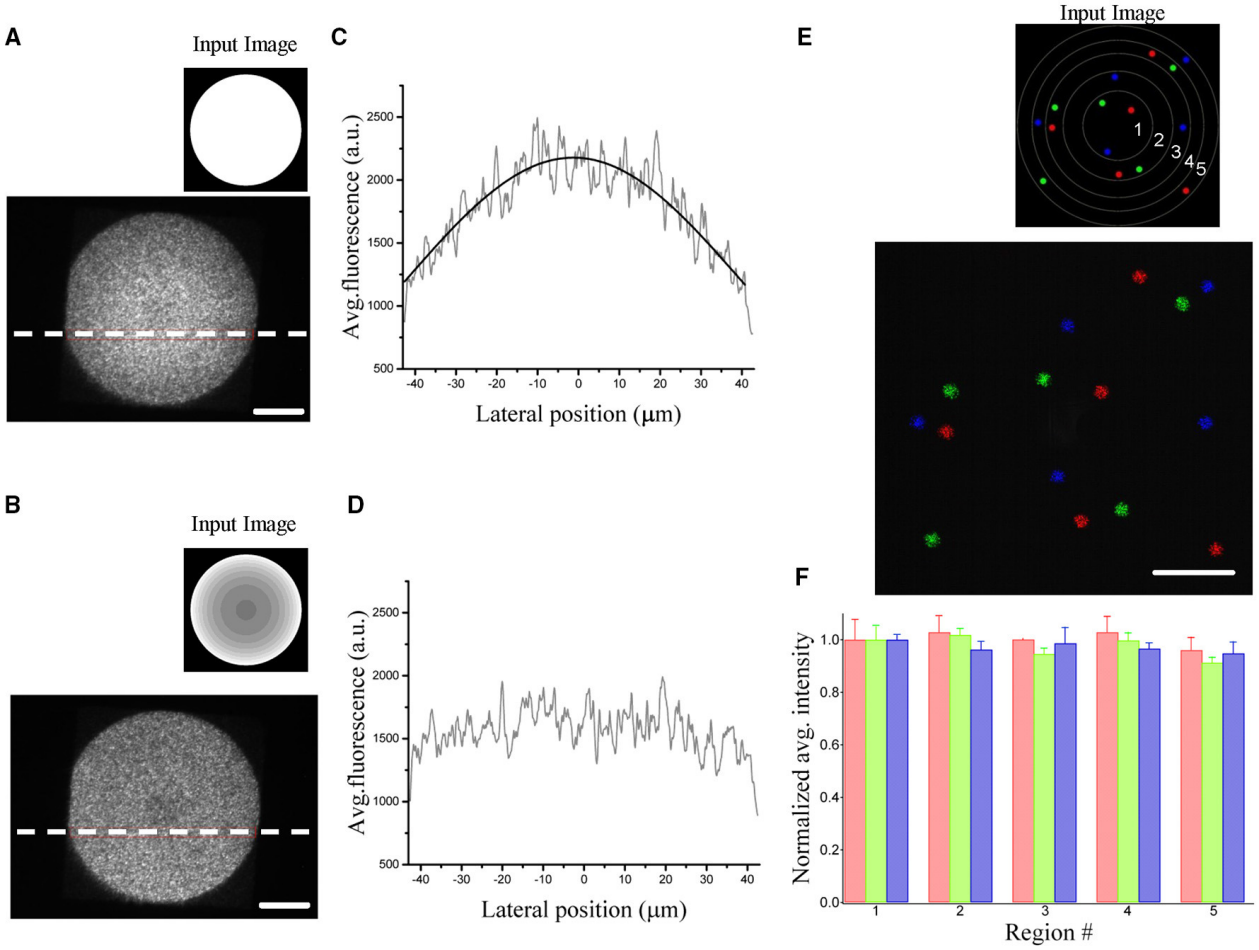
**Demonstration of diffraction efficiency correction using graded intensity input images. (A)** Spatial variation of the diffraction efficiency visualized by illuminating a uniform rhodamine fluorescent layer by a 90 μm holographic spot (inset: corresponding uniform input image processed by the algorithm to generate the phase input of the SLM). **(B)** The same area is illuminated by giving a corrected input image (inset) to compensate for the inhomogeneous diffraction efficiency. **(C)** Average fluorescence intensities along the horizontal dotted lines in **(A)**. The intensity profile is fitted by the equation δ(x) = A^*^sinc^2^(αx). **(D)** The same line profile exhibits a flat intensity profile when using a corrected image. **(E)** Fluorescence superposition image of 3 different patterns (color coded) of five 5 μm-spots distributed over the field of excitation. Each pattern light distribution was imaged on a camera placed on an intermediate image plane using the corrected input image in the inset. The regions delimited by the circles correspond to regions of different diffraction efficiencies for the SLM (1:90%,2:80%,3:70%,4:60%,5:50%) **(F)** Bar graph of the average fluorescence over 5 trials of each spot, normalized with respect to the spots in region 1. Data are grouped according to the regions of different diffraction efficiencies and show no dependence of the equalization efficiency on the location in the FOV. Spots in the same region are color coded according to the image in **(E)**. Scale bars on the images: 20 μm.

The value of δ_1*st*_ depends on the spot position in the field of excitation (FOE) and varies as a sinc^2^ function. The highest diffraction efficiency (Phase Spatial Light Modulator LCOS-SLM, 2014) is in the center of the FOE and the lowest (40.5%) at its edges. The position dependent diffraction efficiency can be expressed as a function of the number of steps N of the phase grating (Dammann, 1970)

$$\begin{array}{l} \delta \left(N\right)={sinc}^{2}\left(\frac{1}{N}\right) \end{array}$$

or as function of the coordinates (*x,y*) of the hologram in the sample plane (Oron et al., 2012).

$$\delta \left(x,y\right)=sinc^{2}\left(\frac{\pi f_{2_{d_{SLM}}}}{\lambda f_{1}f_{obj}}x\right)sinc^{2}\left(\frac{\pi f_{2_{d_{SLM}}}}{\lambda f_{1}f_{obj}}y\right)$$

with *f*_1_, *f*_2_ and *f*_*obj*_ the focal lengths of the lenses and objective, d_*SLM*_ the pixel size and λ the excitation wavelength. Figures 2A,C show the resulting inhomogeneities in the intensity obtained by illuminating a rhodamine layer on a large field of view. The image also reveals spatial fluctuations of about 25%. This speckling distribution is an intrinsic limitation of computer generated holography and it is due to the phase discontinuities at the sample plane inherent to the GS algorithm (Golan et al., 2009).

To correct for the non-uniformity of the diffraction efficiency, we weighted the input image to the GS iterative algorithm (Lutz et al., 2008) such that targets occupying low-efficiency regions were brighter than targets generated at higher efficient locations. A uniform excitation field can thus be generated by rescaling the input image to the GS algorithm accordingly to the expression: $P\left(x,y\right)\;=\;P\cdot \frac{\delta_{min}}{\delta \left(x,y\right)}$ which will generate within the FOE a uniform light distribution equal to Pxδ_min_. Figures 2B,D show the corrected pattern of illumination which is now uniform over the whole excited area.

A similar approach can be also used to generate multiple spots of equal intensity. To clarify this procedure, let us consider for simplicity the case of two targets of equal size generated at two positions corresponding to two different diffraction efficiency values, δ_1_ and δ_min_. Before correction, the light intensity *P*_*tot*_ is equally distributed in the input image as

$$\begin{array}{l} P_{tot}=P_{1}+P_{2}\text{ with }P_{1}=P_{2}=P_{tot}/2 \end{array}$$

However, due to diffraction, at the sample plane, the actual intensity repartition between the two holograms will be

$$\begin{array}{l} P_{tot}=P_{1}\delta_{1}+P_{2}\delta_{min}+o.s \end{array}$$

where *o.s* refers to the higher orders of diffraction and is defined by:

$$\begin{array}{l} o.s=P_{tot}-P_{1}\delta_{1}-P_{2}\delta_{min} \end{array}$$

Uniform light distribution is achieved by modifying the light distribution in the input image according to

$$\begin{array}{l} P_{tot}=P_{1}^{\prime }+P_{2}^{\prime }\;with\;P_{1}^{\prime }=\frac{\delta_{min}}{\delta_{min}+\delta_{1}}P_{tot}\;and\;P_{2}^{\prime }=\frac{\delta_{1}}{\delta_{min}+\delta_{1}}P_{tot} \end{array}$$

giving at the sample plane:

$$\begin{array}{l} P_{tot}=P_{1}^{\prime }\delta_{1}+P_{2}^{\prime }\delta_{min}+o.s\text{ and therefore }P_{1}^{\prime }\delta_{1}=P_{2}^{\prime }\delta_{min}, \end{array}$$

Figures 2E,F show the input sources and the corresponding illumination patterns in the case of multiple equally illuminated spots using the described procedure. To investigate the reliability of the equalization, we generated the same pattern 5 times with arbitrary initial phase distribution in the software calculation of the phase profile. The spots were distributed all over the FOE using 3 different patterns (color coded in the Figures 2E,F). The resulting light intensity distribution on the spots, measured by the camera placed at an internediate image plane in the optical path, showed no spatial dependence in the accuracy of the equalization. All spots, independently from their position in the FOE had the same average light intensity as quantified by the bar diagram in Figure 2F.

### Imaging with intensity-graded holograms

Following the same principle described in the previous section, it is also possible to generate targets with an arbitrary relative light distribution. Let us call α the proportionality factor between the light intensities in 2 points and $P_{1}^{\prime \prime }$ and $P_{2}^{\prime \prime }$ the light intensities that take into account two corrections: the diffraction efficiency and the graded intensity.

The light distribution in the input image will verify in this case:

(1) $$\begin{array}{l} P_{1}^{\prime \prime }+P_{2}^{\prime \prime }\;=P_{tot} \end{array}$$

with

$$\begin{array}{l} P_{1}^{\prime \prime }=\alpha P_{1}^{\prime }=\alpha \frac{\delta_{min}}{\delta_{min}+\delta_{1}}P_{tot}\\ P_{2}^{\prime \prime }=P_{2}^{\prime }=\frac{\delta_{1}}{\delta_{min}+\delta_{1}}P_{tot} \end{array}$$

And therefore:

$$\begin{array}{l} P_{1}^{\prime \prime }=\frac{\alpha \delta_{min}}{\alpha \delta_{min}+\delta_{1}}P_{tot}\\ P_{2}^{\prime \prime }=\frac{\delta_{1}}{\alpha \delta_{min}+\delta_{1}}P_{tot} \end{array}$$

**Figure 3:**
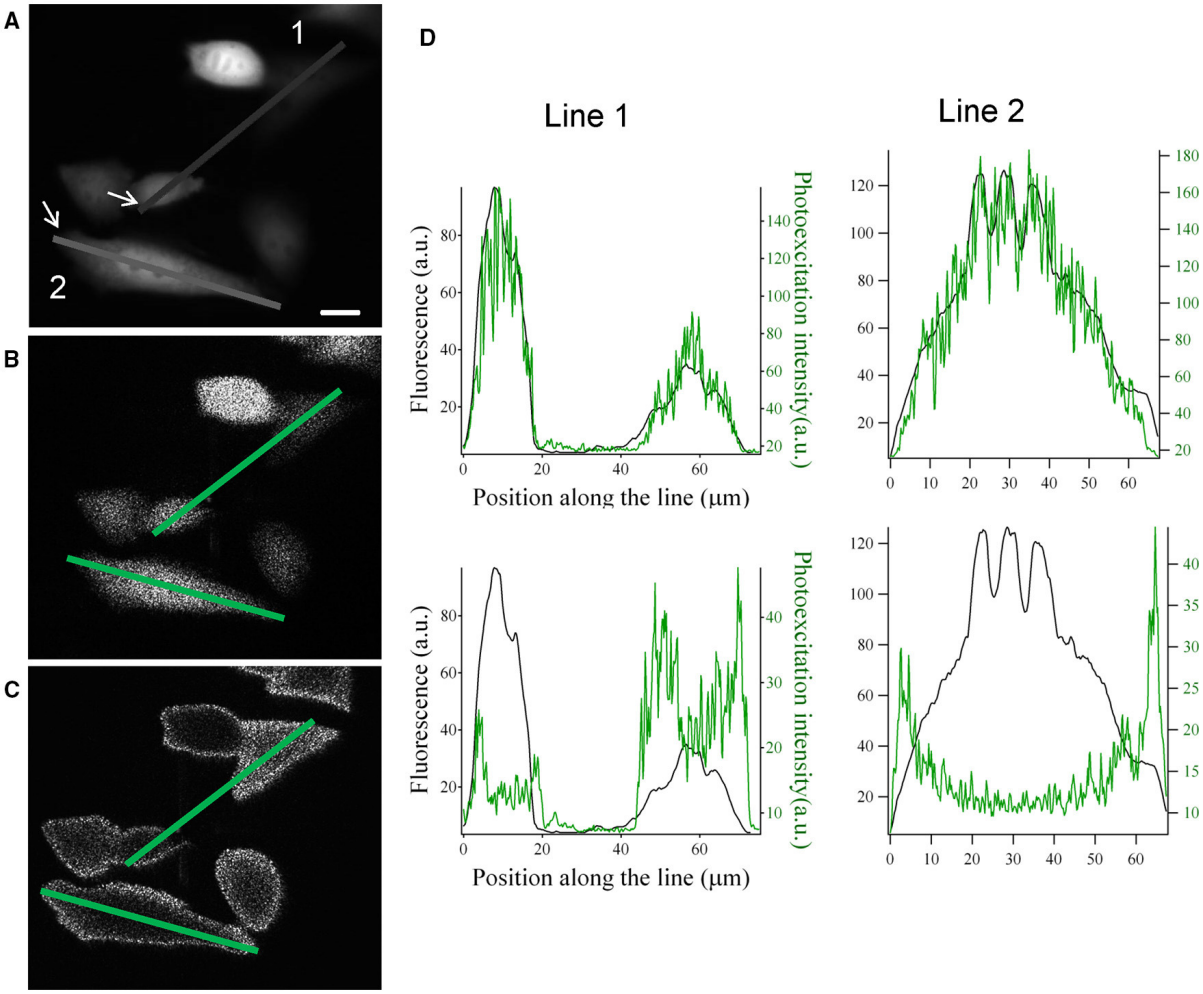
**Graded structured illumination can be used to generate holographic patterns with power distributions that are proportional or inversely proportional to the fluorescence level of a sample. (A)** Epifluorescence image acquired from YFP transfected CHO cells. **(B,C)** images acquired by the camera placed at the image plane with holographic patterns proportional **(B)** and inversely proportional **(C)** to the fluorescence image in **(A)**. Scale bar: 10 μm. **(D)** line profiles along the two lines drawn in panel **(A–C)**. The position along the line is calculated starting from the arrows in panel **(A)**. Top: comparison of the line profiles from images in **(A)** (black line) and **(B)** (green line). Bottom: comparison of the line profiles from images in **(A)** (black line) and **(C)** (green line).

These modifications on the SLM input image can be used to generate intensity-graded holograms where the illumination at the sample plane scales according to the fluorescence distribution, F(x,y) of a biological image. As shown in Figure 3, light distribution can be rescaled either proportionally or inversely proportionally to the fluorescence distribution in the image.

In the case of hologram proportional to the fluorescence image, the light intensity of a point (x,y) in the sample plane *P*_*o*_(*x, y*) will follow:

$$\begin{array}{l} P_{0}\left(x,y\right)=P_{min}+\frac{F_{min}-F_{max}}{P_{min}-P_{max}}\left(F\left(x,y\right)-F_{min}\right) \end{array}$$

In the case of hologram inversely proportional, the light intensity will follow:

$$\begin{array}{l} P_{0}\left(x,y\right)=P_{max}+\frac{P_{max}-P_{min}}{1/F_{min}-1/F_{max}}\left(\frac{1}{F\left(x,y\right)}-\frac{1}{F_{min}}\right) \end{array}$$

With *P*_*max*_ and *P*_*min*_ being respectively the maximum and minimum target intensity in the sample plane, and *F*_*min*_ and *F*_*max*_ the maximum and minimum value of the fluorescent signal in the fluorescent image. In Figures 3A–C we show an example of this application based on the image of fluorescently transfected CHO cells. The hologram with graded intensity is displayed on a camera placed at an intermediate image plane (Figure 1). Here we can see a good spatial correspondence of the original fluorescent signal and the holographic photostimulation with a resolution of a few microns (Figure 3D).

### Generation of graded holograms for photocurrent equalization

Finally, we demonstrated the use of graded intensity holograms to equalize photocurrents in ChR2-YFP expressing cells.

In this case, designing a suitable algorithm for graded illumination required at first to assess the relationship between fluorescence intensity and photocurrent.

**Figure 4:**
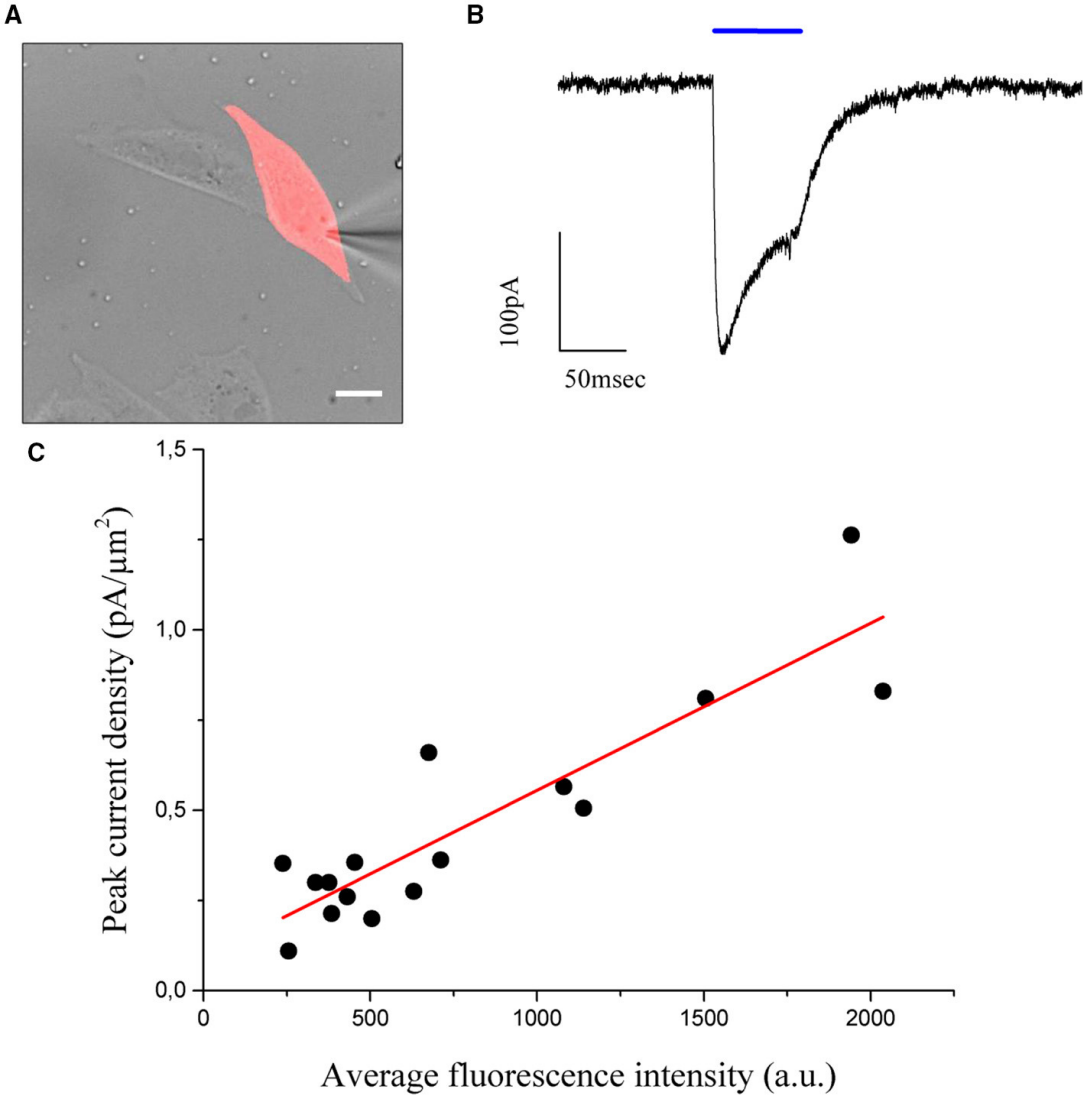
**CHO cells are transfected using the ***cell filling*** plasmid AAV-hSyn-hChr2 (H134R)-p2A-eYFP so that the fluorescence level of the cells is proportional to the density of Chr2 channels**. **(A)** Transmitted light image of transfected CHO cells numerically superposed to the holographic photostimulation pattern (light red). Scale bar: 10 μm. **(B)** Sample current evoked by a holographic photostimulating laser pulse (50 ms duration, 0.05 μW/μm^2^ intensity, λ = 405 nm) illuminating the shape in panel **(A)**. **(C)** Peak current, divided by the stimulated area (current density), is shown to be a linear function of the average fluorescence level using *n* = 16 cells. Experimental data are fitted by a linear function S = aI+b (Pearson's *r* = 0, 896).

To this end, a shaped illumination covering the whole cell body was generated on the base of the transmitted light or epi-fluorescence image (Figure 4A) and used to photostimulate ChR2 expressing cells (15 to 30 ms pulse duration; excitation wavelength 405 nm; excitation density: 0,05 μW/μm^2^) selected to cover a broad range of expression levels. Photo-evoked currents were measured by holding the cells at −40 mV under voltage clamp conditions (*n* = 16 cells) in the whole-cell patch clamp configuration. Upon laser light illumination the cells exhibited a typical photocurrent trace, which showed a fast rise and a slower desensitization phase (Figure 4B) (Nagel et al., 2003). After illumination was discontinued, the photocurrent decayed exponentially to baseline with a time constant τ_off,_ which represents the mean lifetime of the open channels.

The values of photocurrents, measured at the peak after light onset and normalized to the cell surface, were plotted as a function of the corresponding average fluorescence intensity (Figure 4C) and confirmed the expected (Prakash et al., 2012) linear relationship between channel expression and fluorescence levels (coefficients of the linear fit: slope m = 46 ± 6 × 10^−5^ pA/μm^2^ x a.u.; intercept b = 0.09 ± 0.06 pA/μm^2^; Pearson's coefficient for the goodness of the fit: 0.896). Using these results, we proposed to equalize photocurrents in cells with different expression levels by generating holographic spots scaled on the basis of the fluorescence intensity: cells with dimmer fluorescence correspond to lower channel expression and would be excited with higher intensity holograms with respect to brighter cells having higher channel expression. If photocurrents scaled linearly with excitation density, we could simply generate gradient intensity holograms where light intensity scales in an inversely proportional manner with respect to the fluorescence intensity. However this approximation will not take into account the likely situations in which the dependence on excitation light is non-linear (as it is for instance the case for saturation). We needed therefore to first derive the analytical expression describing the dependence of photocurrent on excitation power.

**Figure 5:**
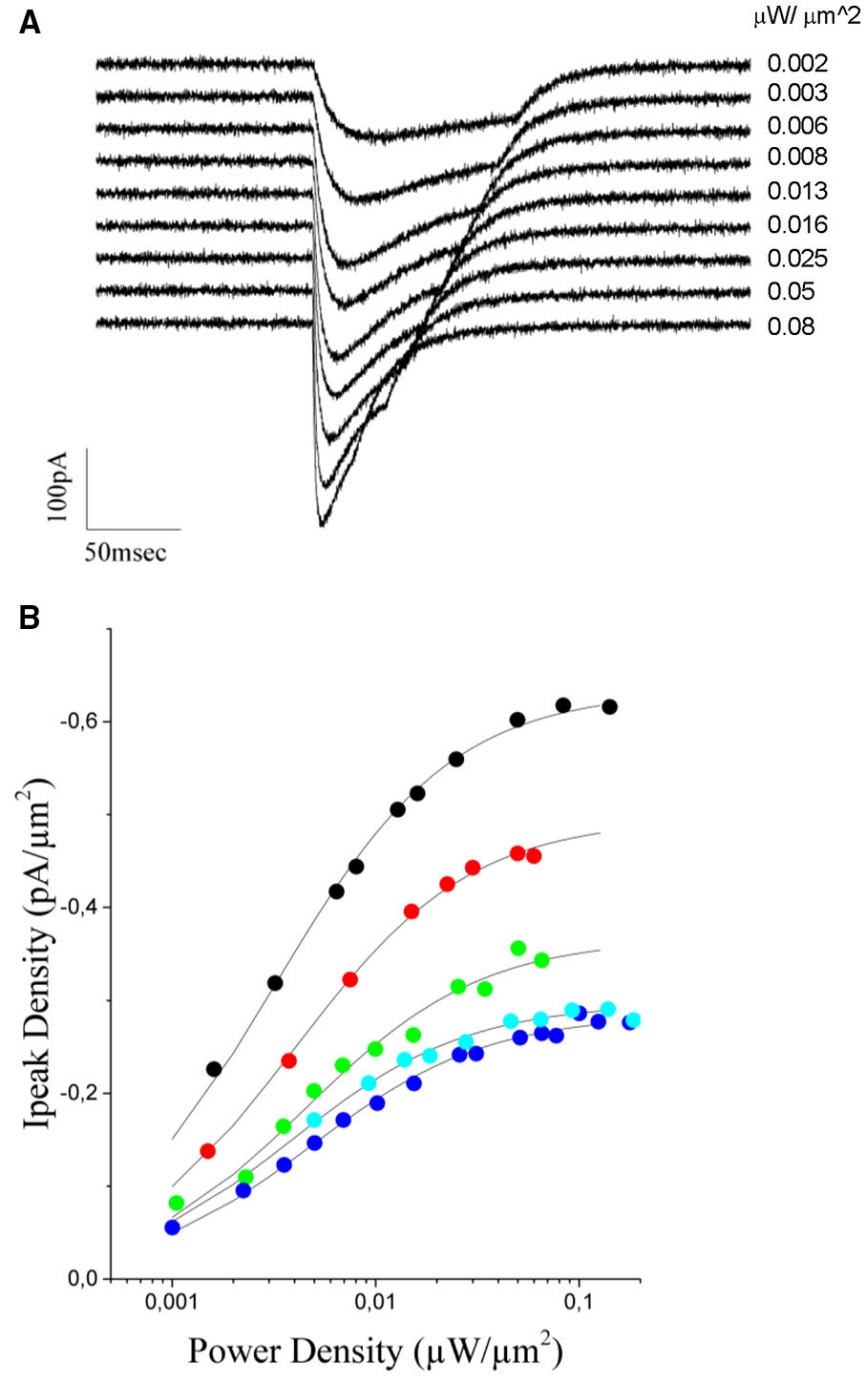
**Power dependence of the ChR2 photoevoked currents in CHO cells expressing the opsin. (A)** Currents evoked in a CHO transfected cell with increasing light intensities (indicated to the right of the trace, the traces correspond to black dots in **B**). **(B)** The peak current density is plotted a function of the excitation intensity for 5 different cells. A Michaelis-Menten equation is used to fit experimental data (solid lines). The average k_D_ value obtained from the fit = 0.004 ± 0.0006 μW/μm^2^.

To this end, we measured (*n* = 5 cells) light evoked currents at different excitation densities (from 0.001 to 0.11 μW/μm^2^; laser pulses from 130 to 20 ms duration) (Figure 5A) and plotted the value of the peak current as a function of the illumination power (Figure 5B). As already reported for this opsin (Rickgauer and Tank, 2009; Asrican et al., 2013), both peak current and rise time increased with illumination power and reached saturation values corresponding to the situation in which a maximum of channels within the illumination volume are open (Figure 5B).

The photocurrent time-dependence of ChR2, under continuous illumination, can be described with a model including two parallel photo-cycles with two connected open (O1 and O2) and closed (C1 and C2) states (Nagel et al., 2003; Hegemann et al., 2005) (O1 and O2 are populated by light illumination of C1 and C2 where they decay back with two different decay times). O1 can also undergo a slow reversible transition toward O2, while C2 (the dark-adapted ground state) slowly decays to the C1 state (end of the photo-cycle).

During the initial phase of the response, up to the peak, desensitization can be neglected and one can consider a simplified model including only the two sates C1 and O1 (Rickgauer and Tank, 2009). Under this approximation, the peak current would represent the equilibrium between these two states and can be well described by a Michaelis-Menten type equation (Michaelis and Menten, 1913) of the form:

(2) $$\begin{array}{r} I_{peak}\;=\;I_{max}\frac{P}{P\;+\;K_{D}} \end{array}$$

were *k*_*D*_ is the excitation power at which *I*_*peak*_ reaches one half of *I*_*max*_.

Using equation (2), we could obtain a good fit of the experimental data using an average value of *k*_*D*_ = 0,0040 ± 0.0006μW/μm^2^ (*n* = 5 cells, Figure 5B). This value and equation (2) were then used to build up the graded input source for the GS algorithm that enables equalization of photocurrents. Precisely, let us assume the case of two cells with a fluorescence intensity equal to F_1_ and F_2_, with F_2_ < F_1_. The ratio ν = F_1_/F_2_ corresponds also to the ratio between the corresponding peak currents (Figure 4C), that is ν = F_1_/F_2_ = *I*_*max*1/_ *I*_*max*2_. Using this value and equation (2) we can derive the scaling factor:

(3) $$\begin{array}{r} R=P_{2}/P_{1}=\left(1\;-\;\beta \right)\frac{\nu }{\left(1\;-\;\beta *\nu \right)},\;where\;\beta =\frac{P_{1}}{\left(P_{1}+K_{D}\right)} \end{array}$$

For cells lying at spatial positions corresponding to different diffraction efficiency values, δ_1_ and δ_2_, equation (3) becomes

(4) $$\begin{array}{r} R=P_{2}/P_{1}=\frac{\left(1-\beta \right)\cdot \nu }{1-\beta \cdot \nu }\cdot \frac{\delta_{1}}{\delta_{2}} \end{array}$$

This way, based on the fluorescence image and knowing the value of K_D_ and the reference power P_1_, it is possible to appropriately rescale the relative power distribution and equalize the responses.

**Figure 6:**
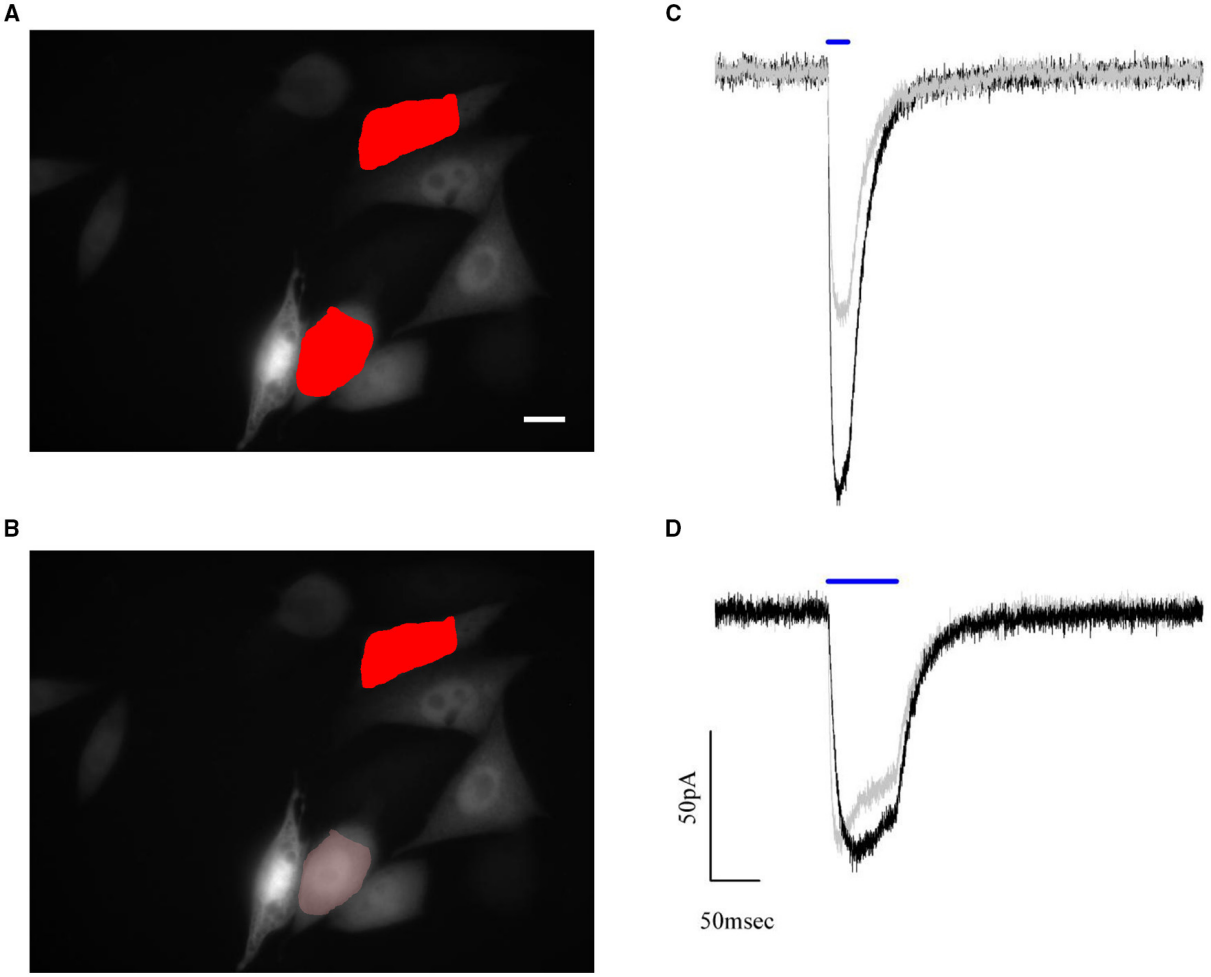
**Automated compensation for uneven expression levels of cells by graded holographic photostimulation**. Epifluorescence image of the YFP signal of ChR2-expressing CHO cells and numerically superposed input shapes on two recorded cells under **(A)** holographic equal light illumination and **(B)** holographic graded light illumination. Scale bar: 10 μm. **(C,D)** Example of photocurrents induced during a paired whole-cell recording of the two photostimulated cells. The photocurrent traces, initially of different amplitude **(C)** due to different expression levels, are brought to the same peak value by reducing the intensity of light sent on the brighter cell **(B)**. This intensity lowering is automated in the software according to the fluorescence levels of the epifluorescence image.

To test the performances of the graded algorithm we used ChR2 expressing CHO cells. A pair of cells with different fluorescence intensity was selected within the available field of view. At first, photocurrents where measured in response of two excitation shapes of equal area and intensity, which we set close to the saturating value (0.05μW/μm^2^; Figure 6A). Under this illumination condition, the difference in the recorded photocurrents reflects the difference in channel density (expression level) on the cell membrane (Figure 6C).

Next, we achieved equalized peak currents by generating a graded hologram producing on the dimmer cell an illumination shape kept at 0.05μW/μm^2^ while rescaling the shape's intensity on the brighter one according to equation (4) (Figures 6B,D).

**Figure 7:**
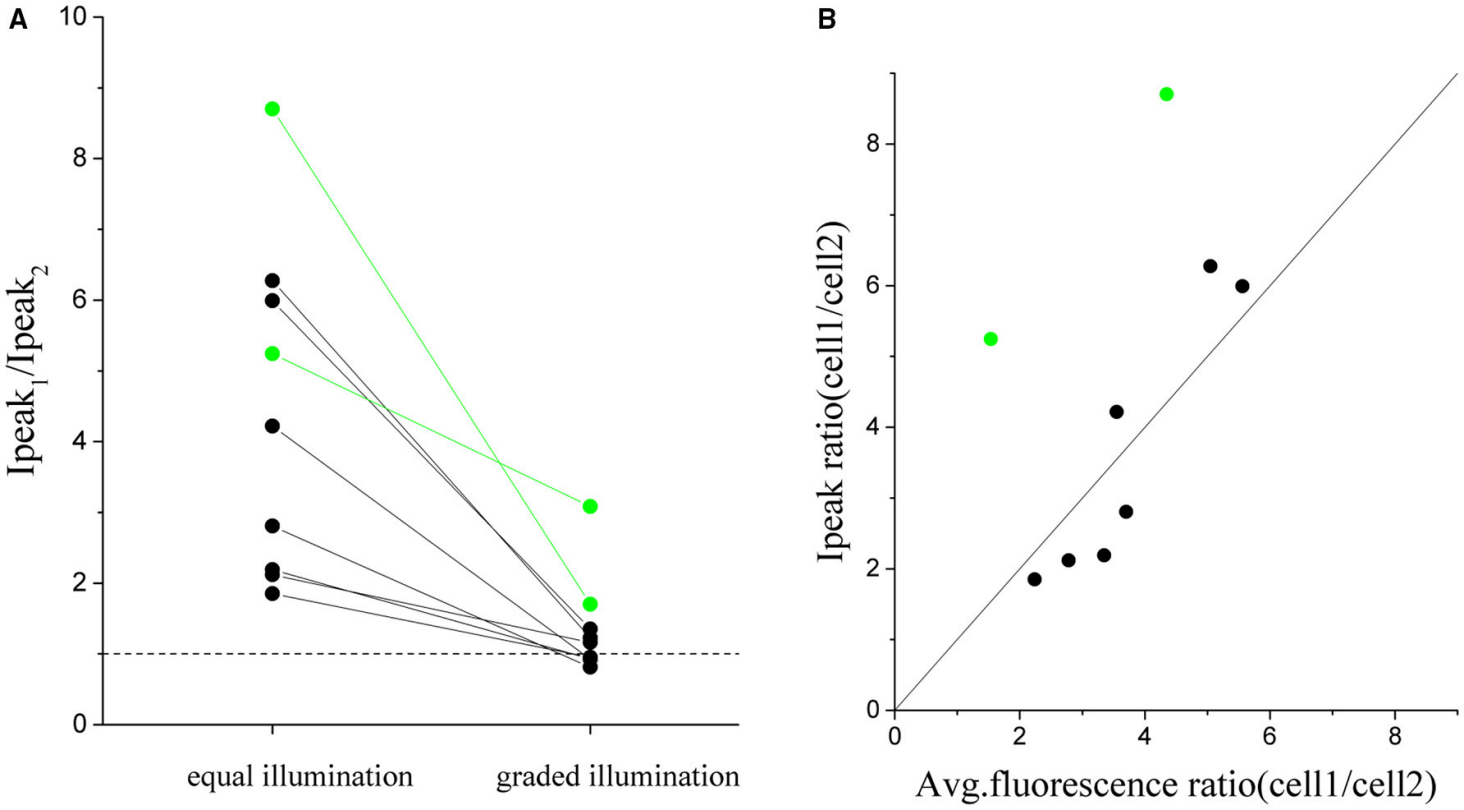
**Cumulated results demonstrating the equalization of the peak current by graded intensity photostimulation**. The ratio between peak currents evoked with illumination of equal intensity is compared to the case of intensity-graded illumination **(A)** for *n* = 9 pairs of differently expressing cells (where cell_1 is the more expressing one) (avg. ratio of equal stimulation peak currents = 4 ± 2; avg. ratio upon graded stimulation = 1.3 ± 0.7). **(B)** Plot of the corresponding ratio of peak current vs the ratio of fluorescence detected for all couples of cells. Superposed in black the identity line (y = x). The two pairs of cells shown in green in the plots are cells in which the ratio between fluorescence and currents did not correspond and for which the protocol gave a less good equalization.

In order to quantify the robustness of this approach we repeated the same experiment of Figure 6 on 9 pairs of cells with an intensity peak ratio, before equalization, ranging from 1.8 to 8.8 (Figure 7A). The ratio after correction goes to 1 in all cases except than for two pairs (green dots). These two cases corresponded to cell whose dependence of photocurrent on fluoresce intensity deviated from the expected linear dependence (Figure 7B). The algorithm could then just reduce the difference between the currents but not completely compensate for it.

## Conclusions

We have shown an optical system enabling the generation of intensity-graded holograms. The GS algorithm intrinsically allows for the capability of generating phase profiles with non-uniform input sources that will result in a light distribution on the image plane with graded intensity.

At first we used graded-intensity holograms to compensate the position dependent diffraction efficiency of LC-SLMs by weighting target intensity input to the GS algorithm in such a way that targets occupying low-efficiency regions at the side of the field of view were brighter than targets positioned at the more efficient central zone. The correct calculation of the relative intensities in the input source enabled reaching uniform light distribution on the entire field of view.

We then show that intensity-graded holograms could be used to rescale the illumination intensity proportionally or inversely proportionally to the fluorescence distribution of the targets. The lateral resolution of the graded hologram is only limited by the intrinsic stochastic distribution of the speckles. In our example, using an upright microscope with a high NA 63x water-immersion objective we could achieve micrometric lateral resolution.

Finally, we demonstrated that intensity-graded holograms could be used to equalize photocurrents in cells expressing ChR2 at different levels. Given the relative density of channels and using an analytical equation for the dependence of the channel on the intensity of light excitation, we could calculate how to correctly weight light distribution in the input source to the GS algorithm in order to obtain the same current amplitude from cells with different levels of expression. To quantify the relative density of channels in the cells, we used a cell-filling opsin construct, where the emission intensity from the fluorescent reporter (YFP) is proportional to the number of channels. To derive the analytical equation to fit the dependence of the peak current on the intensity of photo-stimulating light we used a Michaelis-Menten equation. The equation well fits the experimental data, and it is characterized by the only parameter K_D_. We expect other opsins to have the same dependence upon light stimulation, differing only in the value of K_D_. Experiments using others opsins will thus only necessitate to firstly determine the K_D_ of the opsin under the specific illumination condition. Graded intensity holograms could be also used to compensate other sources of excitability heterogeneity such as when multiple targets lay at different depths in scattering tissue. It could also be used to compensate for difference in structure's input resistance, determined by diameter, surface to volume ratio, and composition of endogenous channels. Using intensity-graded holograms will also permit to generate arbitrary activation patterns in a neuronal network using optogenetics or caged compounds (Zahid et al., 2010). For fast functional imaging using holographic illumination of multiple regions of interest, using graded light illumination will enable to direct more light onto dim regions with respect to more fluorescent structures, thus achieving uniform resting fluorescence and optimal SNR, and consequently, a maximization of the temporal resolution (Foust et al., 2015). Illuminating the sample with a light which is inversely proportional to the actual fluorescence emitted by the sample should allow to increase the signal to noise ratio of smaller structures like thin dendrites, spines, axons and boutons. This could be a useful tool in Ca^2+^ imaging or voltage sensitive dye experiments, in which inversely graded illumination should allow to simultaneously record fluorescence variations in thin structures as well as in the soma and primary dendrites.

## Author contributions

RC, OA, and VE: Designed the experiments; OA: Built up and characterized the optical system; RC: Performed the electrophysiological experiments, data analysis and theory for ChR2 photocurrent equalization; MG: Participated to the optimization of the optical system; VD: Developed the software for the generation of graded intensity pattern; VE, RC, and OA: Wrote the manuscript (with contributions from MG); VE: Supervised the project.

### Conflict of interest statement

The authors declare that the research was conducted in the absence of any commercial or financial relationships that could be construed as a potential conflict of interest.
