# Supplementary material for: Computer Generated Holography with Intensity-Graded Patterns
Source: Front Cell Neurosci. 2016 Oct 17;10:236. doi: 10.3389/fncel.2016.00236 (PMC5065964; doi:10.3389/fncel.2016.00236)
Supplement: Supplementary file 1 [file Image1.PDF]

Supplementary Material

Computer generated holography with intensity-graded patterns

R. Conti[1], O. Assayag[1,#], V. de Sars[1], M. Guillon[1] and V. Emiliani[1*]

[1]Wave front engineering microscopy group, Neurophotonics laboratory CNRS UMR8250,

University Paris Descartes, Paris, France

# present address: Intelligent Imaging Innovations GmbH

Königsallee 9-21, D-37081 Göttingen, Germany

*** Correspondence:**
Valentina Emiliani
valentina.emiliani@parisdescartes.fr

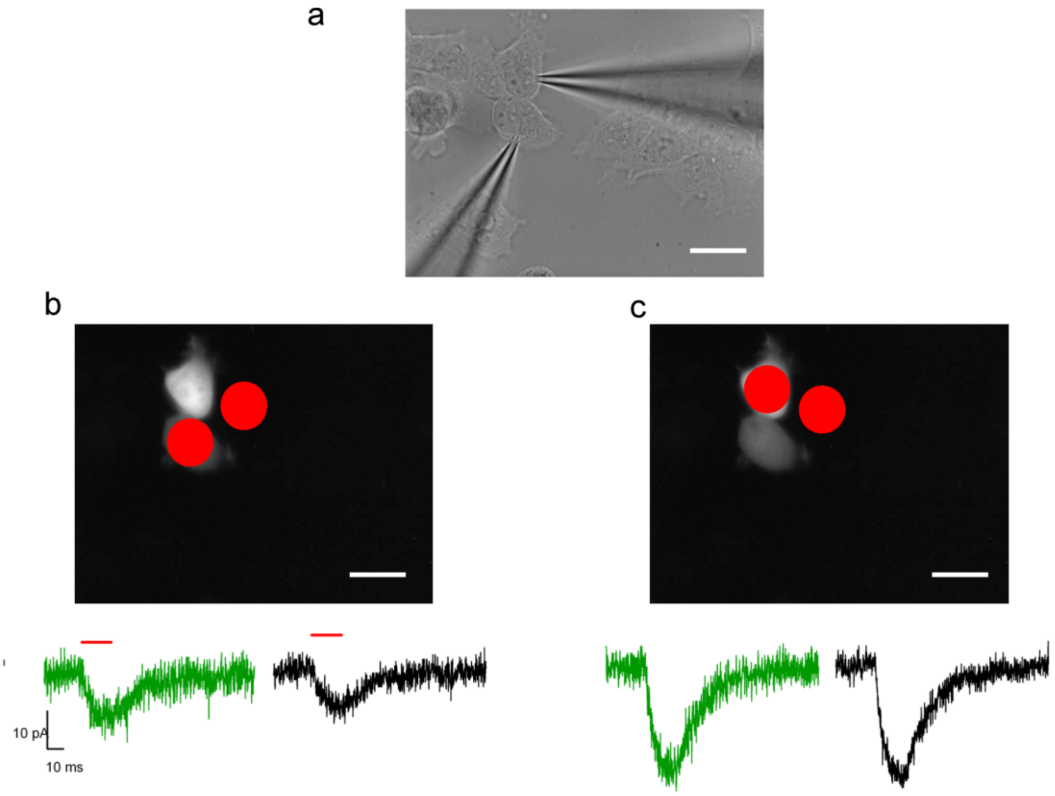

Conti et al. Suppl. Figure 1

Supplementary Figure 1

Gap junctions between HEK cells in a culture dish impair quantitative electrical measurements of photocurrents evoked by holographic photostimulation. a) IR image of two HEK cells in a paired recording configuration. b) Top: Fluorescence image of the YFP expressing CHO cells with superposed pattern of photstimulation (one dot covers the weakly expressing cell and the other is placed on a region void of cells). Bottom: corresponding photoevoked currents (green for the weakly expressing cell, black for the high expressing cell) are of almost equal intensity in the two recorded cells, demonstrating a very high degree of electrical connection. c) Same as in b, but with photostimulation of the brighter cell. In this case the same type of crosstalk, leads to the paradox of having a greater response in the weakly expressing cell when the adjacent one is photostimulated than under direct photostimulation. Scale bars on the images: 20μm.
